# PRELIMINARY DATA ON LIVER TRANSPLANTATION IN HYDATIDOSIS DISEASE

**DOI:** 10.1590/0102-672020210002e1667

**Published:** 2022-06-24

**Authors:** Alexia Rangel de CASTRO, Elodie Bomfim HYPPOLITO, José Telmo VALENÇA-JÚNIOR, Gustavo Rego COELHO, José Huygens Parente GARCIA

**Affiliations:** 1 Federal University of Ceará, Medical School, Fortaleza, Ceara - CE, Brazil ;; 2 Hospital Universitário Walter Cantídio, Liver Transplant Unit, Fortaleza, Ceara - CE, Brazil ;; 3 Federal University of Ceará, Pathology and Legal Medicine Department, Fortaleza, Ceara - CE, Brazil ;; 4 Federal University of Ceara, Department of Surgery, Fortaleza, Ceará - CE, Brazil.

**Keywords:** Echinococcosis, Hepatic, Liver Transplantation, Liver Diseases, Parasitic, Equinococose Hepática, Transplante de Fígado, Hepatopatias parasitárias

## Abstract

Hydatidosis is a rare and endemic parasitic disease in Brazil that causes the proliferation of cysts mainly in the liver, leading to many complications, such as compression of vessels and biliary ducts, liver failure, portal hypertension, and cirrhosis. The treatment of choice is the resection of the lesions combined with albendazole therapy. This disease is a rare indication for liver transplantation, a feasible treatment option in more advanced stages.

**AIM:**

The purpose of this study was to describe two cases of patients from northern Brazil who underwent liver transplantation due to hepatic hydatidosis.

**METHODS:**

This is a retrospective study with data collected from medical records.

**RESULTS:**

Case 1: A 51-year-old female patient presented pain in the right hypochondriac, dyspepsia, consumptive syndrome, and obstructive jaundice, with a previous diagnosis of Caroli’s disease with no possibility of surgical resection and a MELD score of 24. She underwent liver transplantation, and the anatomopathological result demonstrated hydatidosis. Case 2: A 52-year-old female patient presented multiple episodes of cholangitis in 30 years, with three liver resections and clinical treatment with albendazole for hydatidosis. She underwent liver transplantation due to recurrent cholangitis with a MELD score of 20. Both patients underwent post-transplant clinical therapy with albendazole, had good outcomes, and remain in follow-up without complications after 5 and 96 months, respectively.

**CONCLUSION:**

The patients benefited from the procedure and have a good prognosis due to the absence of metastasis, early reintroduction of antiparasitic drugs, and continuous follow-up.

## RESUMO - RACIONAL

A hidatidose é uma doença parasitária rara, endêmica no Brasil, que causa a proliferação de cistos, principalmente no fígado, levando a muitas complicações, como compressão de vasos e ductos biliares, hipertensão portal e cirrose. O tratamento ideal é a ressecção cirúrgica das lesões combinada à terapia com albendazol. Essa doença é uma rara indicação para o transplante hepático, que é um possível tratamento para estágios avançados.

**OBJETIVO:**

Descrever dois casos de pacientes provenientes da região Norte do Brasil, que foram submetidos a transplante hepático por hidatidose hepática.

**MÉTODOS:**

Estudo retrospectivo com coleta de dados de prontuários.

**RESULTADOS:**

Caso 1: Paciente do sexo feminino de 51 anos, apresentava dor em hipocôndrio direito, sintomas dispépticos, síndrome consumptiva e icterícia obstrutiva, com diagnóstico inicial de doença de Caroli sem possibilidade de ressecção cirúrgica e com MELD 24. Foi submetida a transplante hepático, e o resultado anatomopatológico do explante evidenciou hidatidose. Caso 2: Paciente do sexo feminino de 52 anos, apresentava há cerca de 30 anos, múltiplos episódios de colangite, com realização de três ressecções hepáticas e tratamento clínico com albendazol para hidatidose. Foi submetida a transplante hepático por equinococose alveolar difusa, com situação especial por colangite de repetição, com MELD 20. Ambas fizeram tratamento clínico pós transplante com albendazol, apresentaram boas evoluções e permanecem em acompanhamento sem complicações após 5 e 96 meses, respectivamente.

**CONCLUSÃO:**

As pacientes se beneficiaram do procedimento e têm bom prognóstico, devido à ausência de metástases, reintrodução precoce das drogas antiparasitárias e acompanhamento contínuo.

## INTRODUCTION

Echinococcosis or hydatidosis is a rare and endemic disease caused by the infestation of parasites of the *Echinococcus* genus. There are four recognized species of *Echinococcus*: *Echinococcus multilocularis, Echinococcus oligarthrus*, *Echinococcus granulosus,* and *Echinococcus vogeli*, the latter two being found in Brazil, in the southern and northern regions, respectively*.* The intermediate hosts are rodent species, such as “paca,” a typical animal hunted in northern Brazil, and the definitive hosts are domestic or hunting dogs. Men are accidental intermediate hosts that can be contaminated by ingesting feces containing the parasites’ eggs, usually from dogs that consumed the rodent’s liver contaminated with cysts ^3^ ^,^ ^12^ ^,^ ^13^ ^,^ ^16^. After the ingestion of the eggs, the embryos migrate from the intestines to the liver, forming primary gaps, which progress to cysts. This initial process is asymptomatic ^12^.

The proliferation of the hydatic cysts leads to clinical manifestations, and the main symptom is upper abdominal pain. Obstruction jaundice is also very common because of the compression of biliary ducts. However, the cysts may be extrahepatic, causing different symptoms^16^. Other possible manifestations are hepatomegaly, weight loss, anemia, fever, and hemoptysis ^6^.

The lesions can occur in various sites, such as the liver, lungs, brain, spleen, kidney, and heart, with the ability to cause metastasis of a cancer-like nature. The compression or damage to these organs can lead to many complications^18^.

The prognosis is generally poor, and the proliferation of lesions leading to irresectability, chronic liver failure, and other complications may require liver transplantation (LT) as a form of treatment ^10^.

The objective of this study was to record the initial experience of a single service in LT for hydatidosis, containing two case reports of patients from northern Brazil, with their clinical presentation, diagnosis, treatment, evolution, and prognosis.

## METHODS

This is a retrospective study with data collected from medical records of two patients who underwent LT due to hydatidosis, whose case reports have been approved by the Ethics Committee (Hospital Universitário Walter Cantídio/ Universidade Federal do Ceará - 5.187.244) at a single center. These patients continue follow-ups at the same Service.

## RESULTS

These two cases represent approximately 0.1% of the LT performed at this service (2000 LTs from May 2002 to August 2021).

Case 1: A 51-year-old female patient from the city of São Sebastião da Boa Vista, in the state of Pará, northern Brazil.

**Figure 1 -:**
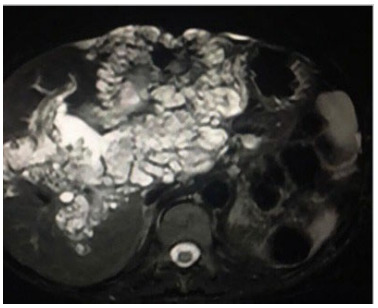
Magnetic resonance imaging of patient in case 1. Axial section, T2-weighted sequence with fat suppression, showing multiple cystic formations with hypersignal, sparse across the liver.

The patient experienced, since 2016, dyspepsia, pyrosis, and intense abdominal pain in the right hypochondriac and epigastric region, once a month, without triggering or relief factors. She underwent abdominal ultrasound and magnetic resonance imaging (Figure 1), which allowed visualization of multicystic liver lesions compromising the entire right lobe and part of the left lobe, suggestive of Caroli’s disease. The patient started presenting weight loss and malnutrition, losing 14 kg in 6 months (27.4% of her usual weight). In 2019, the patient presented floating jaundice, choluria, and pruritus without fever or shivering, leading to evaluation for LT with a MELD score of 24. She underwent LT with a deceased donor in February 2021, due to the impossibility of surgical resection.

The procedure lasted for 7 h, with 6 h and 17 min of cold ischemia and 20 min of warm ischemia. The Piggyback technique was used, with the classical portal and arterial anastomosis ^11^ ^,^ ^15^. Due to the cysts’ invasion of the biliary tract, its anastomosis was performed by Roux-en-Y hepaticojejunostomy. The Institute Georges Lopez-1 (IGL-1) preservation solution was used. The patient received two red blood cells units and one fresh-frozen plasma unit.

**Figure 2 -:**
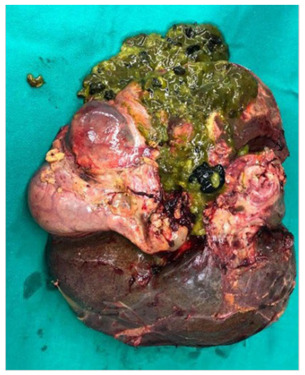
Liver explant of the patient in case 1, with complex structures of *Echinococcus* spp.

Explant biopsy revealed product of total hepatectomy with hepatic echinococcosis, associated with abscesses and epithelioid granulomas. The explant weighted 2.526 g, had 21.9 × 19.2 × 10.5 cm dimensions, and flat brownish external surface (Figure 2). There was moderate macrovesicular steatosis (50% of hepatocytes), moderate perisinusoidal fibrosis, and several cavitations filled by *Echinococcus* spp., characterized by redundant, complex, and birefringent membranes, surrounded by epithelioid granulomas, occasionally forming abscessed areas.

The gallbladder presented echinococcosis and gallstones, with inflammatory infiltrate predominantly neutrophilic, associated with *Echinococcus* spp*.*

Evolution: The patient presented mild graft dysfunction, with good outcome and absence of renal dysfunction. She also presented cholestasis, with resolution in 40 days. The immunosuppressive regimen initiated was tacrolimus and prednisone. She also started the antiparasitic therapy with 400 mg albendazole for 6 months. After 5 months of follow-up, the patient remains alive without any complication.

Case 2: A 52-year-old female patient from the city of Portel, in the state of Pará, northern Brazil.

The patient presented abdominal pain, jaundice, pruritus, fever, and shivering since 1983. She underwent three liver resections and albendazole therapy many times. In 2000, there was another presentation of cholangitis, and the patient was diagnosed with diffuse alveolar hepatic echinococcosis. The disease was bilateral, irresectable, without distant metastasis, and associated with recurrent cholangitis and involvement of the biliary tract, with an indication of LT and a MELD score of 7, which, due to the special situation of recurrent cholangitis, increased the MELD score to 20.

The deceased donor procedure lasted for 6 h and 20 min, with 7 h and 30 min of cold ischemia and 29 min of warm ischemia. The Piggyback technique was used with classical portal, arterial, and biliary tract anastomosis. The patient received two red blood cells units and two fresh-frozen plasma units.

Evolution and treatment: The immunosuppressive regimen initiated was tacrolimus and prednisone, and albendazole was reinstated. The patient had a good immediate outcome. After 96 months of follow-up, the patient remains well and asymptomatic.

## DISCUSSION

Obstructive jaundice is one the most frequent complications, causing choluria, fecal acholia, and pruritus, and it may present as intermittent episodes, accompanied by fever and pain in the right hypochondriac, or cholestasis and long-lasting jaundice. The progression of the cysts may also lead to portal hypertension and cirrhosis ^12^.

However, one-third of the cases are asymptomatic, having an incidental diagnosis. The diagnostic process occurs with the assessment of the patient’s clinical and epidemiological history, as well as the physical examination, imaging studies-mainly abdominal computed tomography and ultrasound, laboratory data, serological tests, and parasitological studies ^5^ ^,^ ^7^.

The patient in case 1 had a pretransplant diagnosis of Caroli’s disease, a congenital condition that causes dilations of the intrahepatic bile ducts, which can lead to recurrent bacterial cholangitis. The correct diagnosis of hydatidosis was possible only after histopathological analyses due to its similarities with the clinical and imaging characteristics of Caroli’s disease. The patient in case 2 had a more typical presentation and a more prominent epidemiological correlation ^8^.

The treatment is based on a multidisciplinary approach, with options that vary from surgical resection of the involved areas, interventional procedures, and LT associated with anti-infective therapy. The approach depends on the size, location, relation to bile ducts and blood vessels, and type of cyst, as well as on the patient’s clinical condition and complications and the surgical team’s experience ^2^ ^,^ ^7^ ^,^ ^18^.

The treatment of choice is the early resection of the hepatic lesions with adjuvant antiparasitic drugs, such as albendazole and mebendazole, which are believed to have parasitostatic effects, slowing down the growth of the masses. The resectability varies from 15% to 87% ^2^.

The anti-infective treatment of choice is albendazole, due to its higher efficacy, bioavailability, and easier administration, with a dose of approximately 15 mg/kg/day. Mebendazole can be used if the patient presents adverse effects or does not tolerate albendazole treatment ^18^.

LT is a good therapeutic option for patients with advanced stages that would not benefit from the cyst’s resection, as well as when there is biliary duct and blood vessels involvement or diffuse hepatic disease, leading to portal hypertension and cirrhosis ^6^. In these cases, the LT combined with the antiparasitic drugs is the only potentially curative treatment, allowing satisfactory long-term results ^14^.

Some factors may lead to a worse prognosis after the procedure, such as the presence of metastasis, which may suffer rapid growth with the immunosuppression therapy, and the delay in reintroducing the antiparasitic drugs. Therefore, to maximize the procedure’s success, previous evaluation of the presence of metastasis in patients with the potential to undergo LT and the early reintroduction of the antiparasitic drugs after the transplant is essential, as well as an adequate patient follow-up ^4^ ^,^ ^17^.

A review paper demonstrated that LT granted more than 10 years of survival in patients with hydatidosis, qualifying it as a feasible treatment for advanced disease, despite the risk of recurrent parasitic lesions induced by immunosuppression therapy ^1^.

In Brazil, there is one case reported of LT in hepatic hydatidosis, in a 48-year-old male patient also from Northern Brazil, who presented recurrent episodes of cholangitis, as the case 2 reported in this article. On the 31st postoperative day, the patient died due to a pulmonary embolism, and the autopsy showed an apparent healthy hepatic graft ^6^.

The continuous use of the antiparasitic treatment as anti-relapse chemotherapy can prevent metastasis growth after the LT and the initiation of the immunosuppressive therapy ^19^.

The survival rate after LT has been reported to be 71% in 5 years, with a 58% rate of disease-free survival. The mortality in the early stages after LT occurs more frequently due to sepsis, and in the late stages, due to recurrence, persistent disease, and metastasis, especially to the brain ^4^ ^,^ ^9^.

## CONCLUSION

Hydatidosis is a rare indication of LT in the world. The patients in the cases above benefited from the LT, had good outcomes, and have a good prognosis due to the absence of metastasis, the early reintroduction of antiparasitic drugs, and the continuous follow-up.
